# Butylated Neuropeptide Antagonist Targeting Hypoxia-Induced GRPR Overexpression in Small Cell Lung Cancer

**DOI:** 10.3390/ijms262110786

**Published:** 2025-11-06

**Authors:** Suttikiat Deureh, Amira M. Alghamdi, Ayşe Latif, Kaye J. Williams, Roben G. Gieling, Harmesh S. Aojula

**Affiliations:** 1 Division of Pharmacy and Optometry, School of Health Sciences, Faculty of Biology, Medicine and Health, University of Manchester, Oxford Road, Manchester M13 9PT, UK; suttikiat.deureh@postgrad.manchester.ac.uk (S.D.); ayse.latif@manchester.ac.uk (A.L.); kaye.williams@manchester.ac.uk (K.J.W.); 2 Department of Biochemistry, Faculty of Science, King Abdulaziz University, Jeddah 21589, Saudi Arabia; amaalghamdi1@kau.edu.sa; 3 School of Geography and Natural Sciences, Faculty of Science and Environment, Northumbria University, Newcastle upon Tyne NE1 8ST, UK; roben.gieling@northumbria.ac.uk

**Keywords:** small cell lung cancer, GRPR, hypoxia, neuropeptide antagonist, targeted therapy

## Abstract

Small cell lung cancer (SCLC) is an aggressive neuroendocrine tumour with limited treatment options and a poor prognosis. Hypoxia, a hallmark of solid tumours, contributes to therapeutic resistance and tumour progression. Gastrin-releasing peptide receptor (GRPR) is known to be overexpressed in SCLC; however, its regulation under hypoxic conditions is not well described. In this study, we demonstrate that hypoxia significantly enhances GRPR expression in SCLC cell lines, COR-L24 and DMS79, as confirmed by Western blot, immunofluorescence, and flow cytometric analysis of binding with fluorescein isothiocyanate–labelled bombesin (BBN-FITC), a known GRPR ligand. To exploit this upregulation, we synthesised a previously discovered butylated neuropeptide antagonist (BU peptide) using a new method of solid-phase peptide synthesis (SPPS) by Boc chemistry and evaluated its therapeutic potential. BU peptide exhibited potent, dose-dependent cytotoxicity in both cell lines, with significantly greater efficacy under hypoxic conditions compared to normoxia. Mechanistic studies revealed that BU peptide inhibits GRP–GRPR-mediated activation of the PI3K/Akt and MAPK/ERK signalling pathways, known to be key regulators of tumour cell survival and proliferation. Moreover, BU peptide induced robust caspase 3/7-mediated apoptosis, especially under hypoxic conditions. These findings suggest that GRPR is a hypoxia-inducible target in SCLC and demonstrate that a synthetically optimised BU peptide antagonist exerts selective efficacy against hypoxic tumour cells, outperforming conventional chemotherapy agents. These findings provide new mechanistic insights into SCLC and suggest translational potential to inform the development of future treatment strategies for this and other hypoxia-driven malignancies.

## 1. Introduction

Small cell lung cancer (SCLC) accounts for approximately 15% of all lung cancers and presents significant clinical challenges due to its aggressive nature, rapid metastasis, and resistance to conventional therapies [1]. One of the critical factors driving the malignancy of SCLC is the hypoxic microenvironment, prevalent in solid tumours due to inadequate vascularisation and rapid tumour growth [2]. Hypoxia induces numerous molecular and physiological changes in cancer cells, including altered receptor expression and increased therapy resistance [3,4].

Gastrin-releasing peptide (GRP), a member of the bombesin-like peptide family, regulates various physiological functions, including gastrin secretion and neurotransmission. GRP induces signalling through its receptor (GRPR), a G protein–coupled receptor (GPCR) that has been implicated in promoting cell proliferation, metastasis, and survival in several cancers such as those originating from the prostate, breast, pancreas and lung [5]. GRPR is known to be overexpressed in many types of cancer under normoxic conditions [5,6]. However, the influence of hypoxia on GRPR expression has not been well-documented, especially in SCLC. Hypoxia is a hallmark of solid tumours and is known to transcriptionally activate key genes such as vascular endothelial growth factor (VEGF) and carbonic anhydrase IX (CAIX). Both are well-established targets of hypoxia-inducible factor-1 (HIF-1) via hypoxia response elements (HREs) in their promoters [7,8,9]. Beyond these canonical markers, other GPCRs involved in cancer progression have also been shown to be hypoxia-responsive. For example, the neuromedin B receptor (NMBR), a bombesin receptor subtype closely related to GRPR, was found to be transcriptionally upregulated under hypoxic conditions via HIF-1α in breast cancer cells [10]. This suggests that bombesin-like GPCRs may share regulatory mechanisms under hypoxia. Based on this, we hypothesised that hypoxia may also enhance GRPR expression in SCLC. This upregulation could render hypoxic tumour regions particularly susceptible to GRPR-targeted therapies. Addressing this knowledge gap is crucial, as hypoxic tumour regions are often associated with poor therapeutic responses and worse clinical outcomes.

Previously, our group has reported a tryptophan prenylated peptide, based on the substance P agonist G (SP-G) sequence, which showed enhanced cytotoxicity against SCLC cell lines: H69 and DMS79, compared to the parent SP-G sequence in vitro [11]. Further optimisation of the N-alkyl substitution on indole ring generated a pentapeptide having D-Trp(N-butyl) residue at the fourth position, herein termed as the BU peptide, with a relatively shortened amino acid sequence compared to SP-G, displayed a further significant enhancement in cytotoxicity in SCLC cells [12].

Targeted therapies utilising peptide antagonists offer several advantages over conventional treatments, including improved specificity, reduced systemic cytotoxicity, and the ability to overcome mechanisms of drug resistance [13,14,15]. Previously made by laborious solution phase chemistry, we now report for the first time the synthesis of BU peptide using solid-phase peptide synthesis (SPPS) method. BU peptide stems from parent peptide SP-G, a known substance P antagonist exhibiting increased antitumor activity linked with higher expression of the GRPR in SCLC [16]. Similarly, BU peptide may selectively target GRPR and inhibit its signalling. Thus, the objectives of this study were threefold: (i) to determine whether hypoxia upregulates GRPR expression in SCLC cells; (ii) to define whether a newly synthesised BU peptide antagonises GRP–GRPR signalling through the PI3K/AKT and MAPK/ERK pathways; and (iii) to evaluate the cytotoxic and pro-apoptotic effects of BU peptide under normoxic and hypoxic conditions.

## 2. Results

### 2.1. Synthesis and Characterisation of BU Peptide

**Figure 1:**
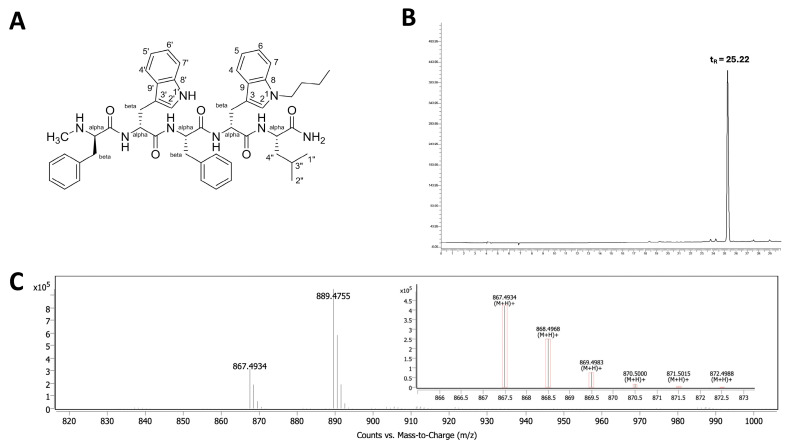
Characterisation of the BU peptide synthesised via SPPS. (**A**) Chemical structure of the pentapeptide C_47_H_54_N_8_O_5_H, featuring N^ind^-butyl modification at the D-Trp residue to enhance GRPR affinity. (**B**) HPLC chromatogram of the purified BU peptide indicating a retention time (t_R_) of 25.22 min, confirming a purity >95%. (**C**) ESI-MS spectrum showing the expected molecular ion peak at *m*/*z* 867.4934 [M+H]^+^ and 889.4755 [M+Na]^+^, consistent with the calculated molecular weight of the BU peptide (866.49).

The butylated neuropeptide antagonist BU peptide was successfully synthesised via SPPS using standard *t*-Boc chemistry (Figure 1A). The peptide sequence, DMePhe-DTrp-Phe-DTrp(N-butyl)-Leu-NH_2_, incorporates an N^ind^-butyl modification at the D-Trp^4^ position, which is considered critical for enhanced cytotoxicity [11,12]. The crude peptide was purified using reverse-phase HPLC, yielding >95% purity with a retention time (t_R_) of 25.22 min (Figure 1B). Electrospray ionisation mass spectrometry (ESI-MS) confirmed the expected [M+H]^+^ molecular ion at m/z 867.49 and 889.4755 [M+Na]^+^ (Figure 1C), in agreement with the theoretical mass of BU peptide (866.49).

### 2.2. Hypoxia Enhances GRPR Expression in SCLC Cell Lines

**Figure 2:**
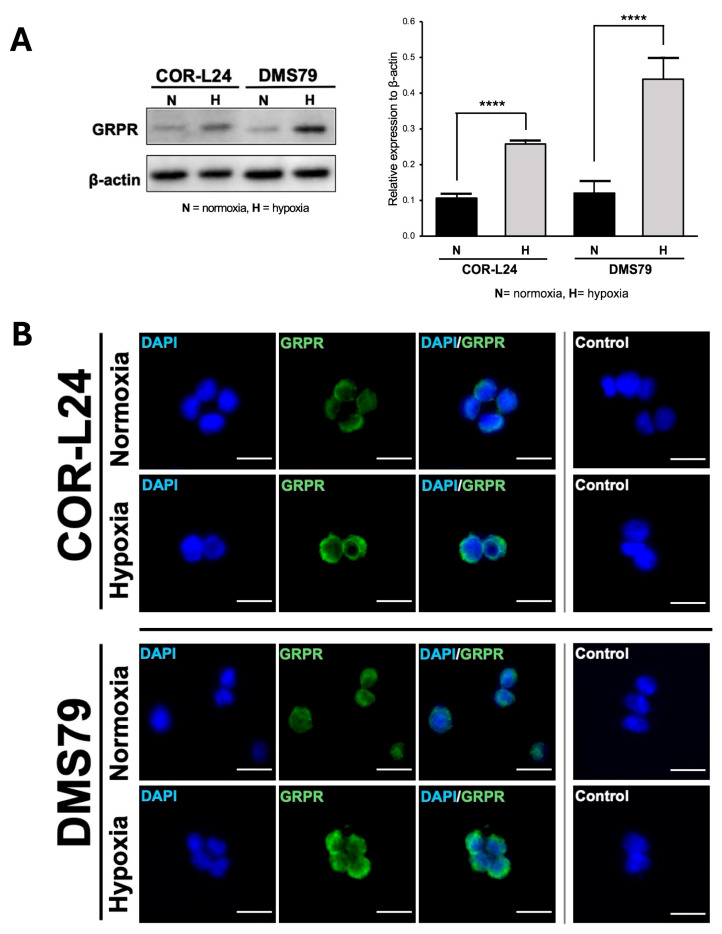
Expression of GRPR in SCLC cell lines under normoxia and hypoxia. (**A**) The quantitative analysis of GRPR expression under hypoxia (0.1% O_2_) and normoxia (20% O_2_) conditions in SCLC cell lines DMS79 and COR-L24, assessed by Western blot. (**B**) The qualitative analysis of GRPR expression using immunofluorescence staining, with DAPI (blue) indicating cell nuclei and GRPR (green) highlighting receptor localisation. Negative control was incubated without GRPR antibody. Scale bar = 50 µm. Data represent mean ± SD from three independent experiments (**** *p* < 0.0001).

**Figure 3:**
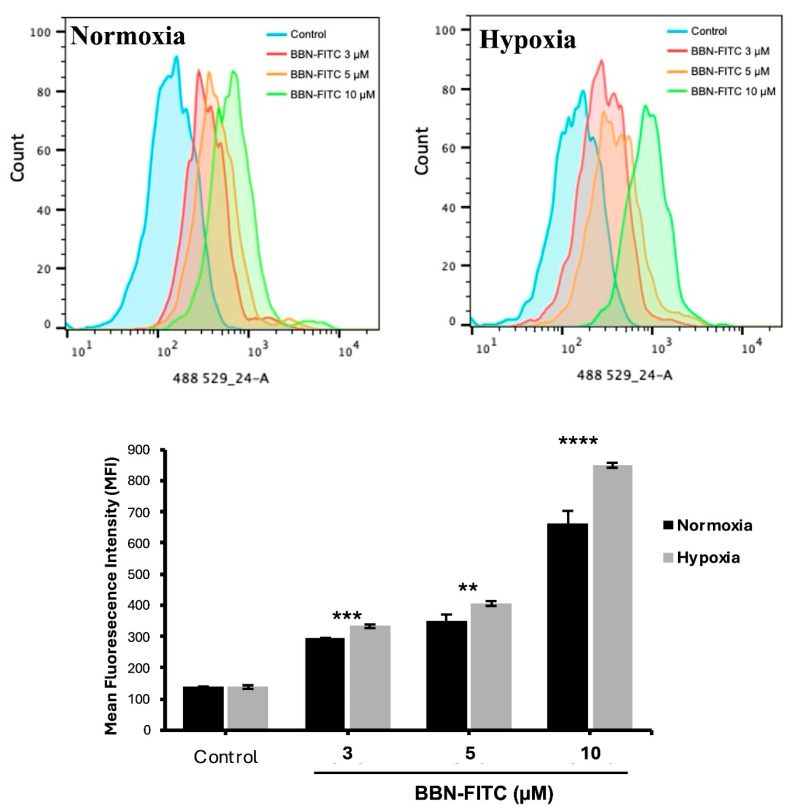
Flow cytometry analysis of BBN-FITC binding to GRPR in SCLC cells under normoxia and hypoxia. Mean fluorescence intensity (MFI) of BBN-FITC binding to GRPR in DMS79 cells cultured under normoxic and hypoxic conditions. Data represent mean ± SD from three independent experiments (** *p* < 0.01, *** *p* < 0.001, **** *p* < 0.0001).

To investigate the relationship between hypoxia and GRPR expression in SCLC, two human SCLC cell lines, COR-L24 and DMS79 were used. COR-L24 is a representative classic neuroendocrine SCLC model, while DMS79 is frequently used to study chemoresistant phenotypes. The expression of GRPR in SCLC cell lines COR-L24 and DMS79 was evaluated under normoxic (20% O_2_) and hypoxic (0.1% O_2_) conditions. Western blot analysis demonstrated that GRPR protein levels were significantly increased under hypoxia compared to normoxia in both cell lines (*p* < 0.0001). Quantitative densitometric analysis, normalised to β-actin, revealed approximately a 2.4-fold increase in GRPR expression under hypoxia in COR-L24 cells and a 3.6-fold increase in DMS79 cells relative to normoxic conditions (Figure 2A). These findings were further supported by immunofluorescence staining, where hypoxic cultures exhibited markedly stronger GRPR-specific fluorescence signals compared to normoxic controls (Figure 2B). To confirm the functional consequence of increased GRPR expression, the binding capacity of bombesin-FITC (BBN-FITC), a GRPR-selective fluorescent ligand, was evaluated by flow cytometry in DMS79 cells only, as these cells had higher GRPR expression levels than COR-L24. The results showed that the mean fluorescence intensity of BBN-FITC binding was significantly increased in hypoxic cells in a dose-dependent manner compared to normoxic conditions, with the most pronounced effect observed at 10 µM BBN-FITC (*p* < 0.0001) (Figure 3). Together, these findings confirm that hypoxia promotes GRPR overexpression at the protein level and enhances functional receptor availability and ligand-binding capacity in SCLC cells.

### 2.3. BU Peptide Attenuates GRP-Induced Phosphorylation of Akt and ERK1/2 in DMS79 Cells

**Figure 4:**
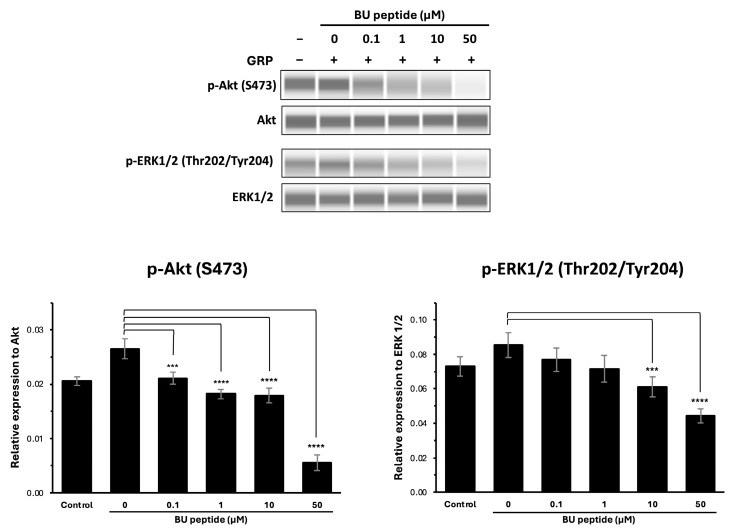
BU peptide inhibits GRP-induced phosphorylation of AKT and ERK1/2 in DMS79 cells under hypoxic condition. Western blot analysis of phosphorylated Akt (p-Akt), total Akt, phosphorylated ERK1/2 (p-ERK1/2), and total ERK1/2 in DMS79 cells treated with increasing concentrations of BU peptide (0–50 µM) for 30 min, followed by stimulation with GRP (100 nM) for 15 min. Control represents vehicle-treated cells without GRP or BU peptide. Densitometric quantification of p-Akt and p-ERK1/2 expression levels normalised to total Akt and ERK1/2, respectively. Data are presented as mean ± SD from three independent experiments (*** *p* < 0.001, **** *p* < 0.0001).

To investigate whether BU peptide interferes with GRP-GRPR-mediated downstream signalling pathways, DMS79 cells were treated with increasing concentrations of BU peptide (0 to 50 µM) for 30 min, followed by stimulation with GRP for 15 min. Phosphorylation levels of Akt (Ser473) and ERK1/2 (Thr202/Tyr204) were evaluated by capillary-based immunoassay (automated Western blot analysis), with total Akt and total ERK1/2 used as loading controls. As shown in Figure 4, GRP stimulation markedly induced the phosphorylation of both Akt and ERK1/2 compared to vehicle-treated controls in the DMS79 cell line. However, pre-treatment with BU peptide led to a dose-dependent reduction in phosphorylation levels of Akt and ERK1/2. Notably, at 10 µM and 50 µM concentrations, BU peptide significantly reduced p-Akt and p-ERK1/2 levels compared to GRP-only treatment (*p* < 0.05), indicating effective inhibition of GRP-induced signalling pathways. These findings suggest that BU peptide can inhibit GRP-mediated activation of the PI3K/Akt and MAPK/ERK pathways in SCLC cells, potentially by interfering with GRP receptor signalling.

### 2.4. Competitive Binding of BU Peptide to GRPR Under Normoxic and Hypoxic Conditions

**Figure 5:**
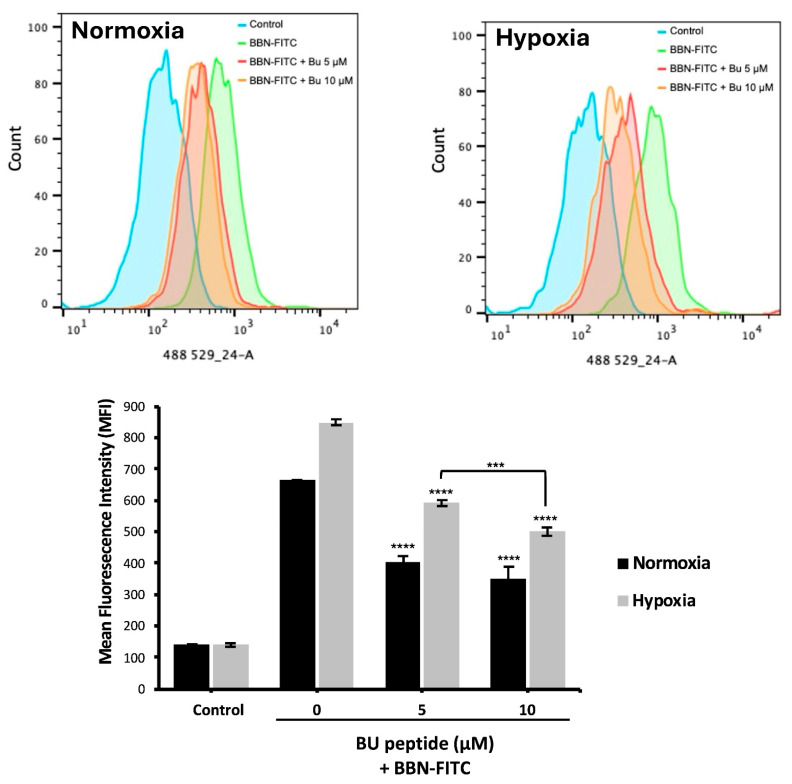
Competitive binding of BU peptide to GRPR under normoxic and hypoxic conditions. DMS79 cells were pre-incubated under normoxia and hypoxia for 24 h, followed by treatment with BU peptide (5–10 µM) for 1 h and incubation with 10 µM BBN-FITC for 30 min. Fluorescence intensity of BBN-FITC was analysed by flow cytometry. Data represent mean ± SD from three independent experiments (*** *p* < 0.001, **** *p* < 0.0001).

To evaluate whether BU peptide binds competitively to GRPR, DMS79 cells were pre-incubated under normoxic and hypoxic conditions for 24 h, followed by treatment with BU peptide (5–10 µM) for 1 h and subsequent incubation with BBN-FITC (10 µM) for 30 min. Flow cytometry was used to quantify the fluorescence intensity of BBN-FITC as an indicator of GRPR binding. As shown in Figure 5, the BBN-FITC fluorescence signal was markedly increased under hypoxia, consistent with elevated GRPR expression. Pre-treatment with BU peptide significantly reduced BBN-FITC fluorescence intensity in both normoxic and hypoxic cells compared to untreated control, confirming receptor competition. Notably, under hypoxia, where GRPR expression is highest, BBN-FITC signal decreased by approximately 10.7% between 5 and 10 µM BU peptide treated, whereas under normoxia, no significant difference was observed between these concentrations. These findings indicate that BU peptide competes with BBN for GRPR binding, with stronger competitive inhibition observed under hypoxic conditions.

### 2.5. BU Peptide Induces Cytotoxicity in Hypoxic SCLC Cells

**Figure 6:**
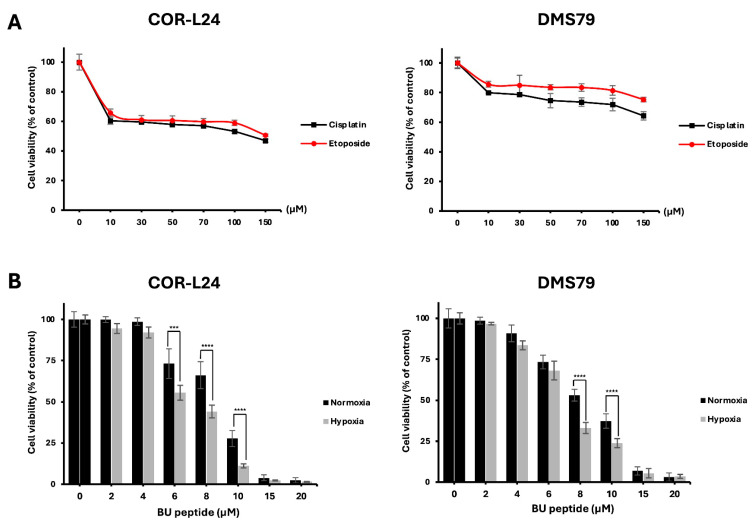
BU peptide exhibits enhanced cytotoxicity under hypoxia compared to conventional chemotherapy in SCLC cell lines. (**A**) COR-L24 and DMS79 cells were treated with cisplatin and etoposide for 24 h under normoxic conditions. (**B**) Cells were pre-incubated under normoxia or hypoxia for 24 h to induce differential GRPR expression, then treated with BU peptide for 24 h. Cell viability was assessed using the CellTiter-Glo assay and expressed as a percentage relative to untreated control cells. Data represent mean ± SD from three independent experiments (*** *p* < 0.001, **** *p* < 0.0001).

The cytotoxic activity of BU peptide was evaluated in COR-L24 and DMS79 cells under normoxic (20% O_2_) and hypoxic (0.1% O_2_) conditions. For comparison, the effect of conventional chemotherapeutic agents, cisplatin and etoposide was first assessed. Both drugs reduced cell viability by approximately 50–80% at concentrations up to 150 µM after 24 h of treatment under normoxia (Figure 6A). In contrast, BU peptide, at much lower concentrations (15–20 µM), achieved a greater reduction in cell viability, indicating superior potency compared to standard chemotherapeutic agents under the same condition of normoxia (Figure 6B). Importantly, BU peptide treatment resulted in a significant, concentration-dependent decrease in cell viability under hypoxic conditions, with a more pronounced effect than that observed under normoxia. These findings suggest that BU peptide not only exhibits enhanced cytotoxic potential over conventional agents but may also selectively target hypoxia-adapted tumour cells, especially in SCLC.

### 2.6. BU Peptide Promotes Apoptosis via Caspase 3/7 Activation

**Figure 7:**
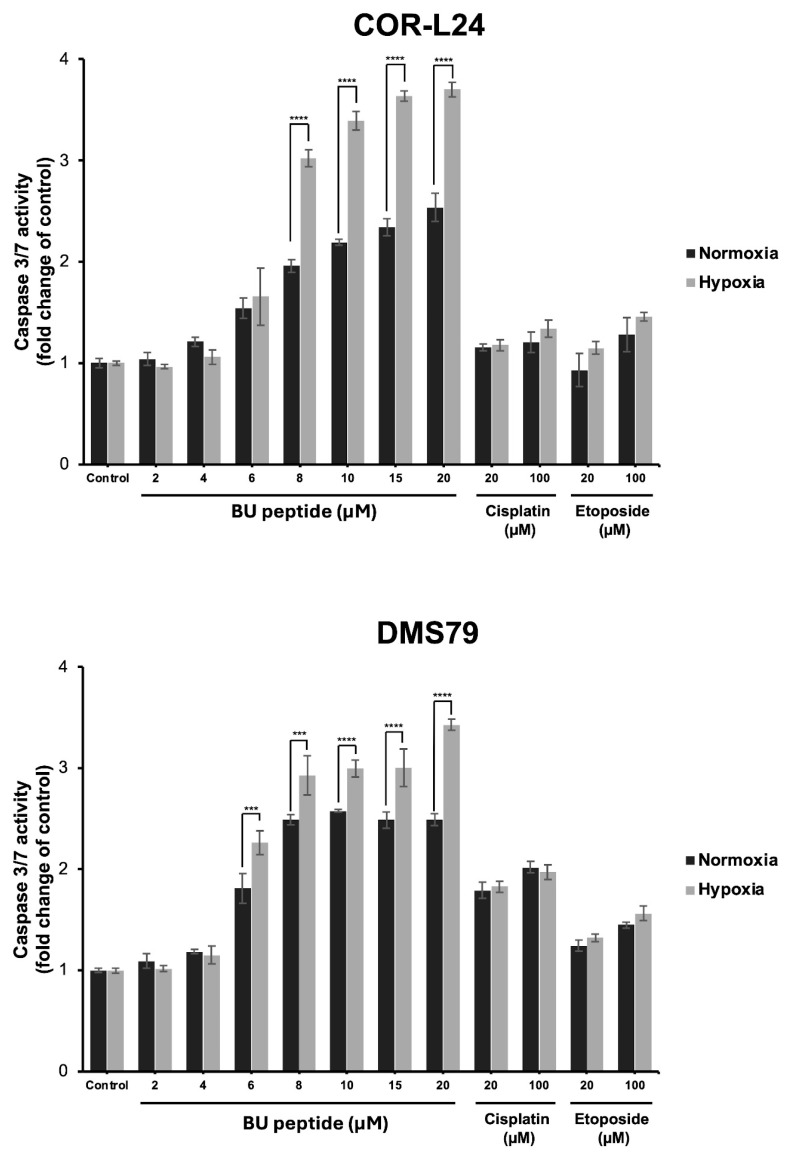
Caspase 3/7 activity in COR-L24 and DMS79 cells treated with BU peptide, cisplatin, and etoposide under normoxic and hypoxic conditions. Caspase 3/7 activity was measured in COR-L24 (top) and DMS79 (bottom) cells following 24 h treatment with BU peptide (2–20 µM), cisplatin, and etoposide (20–100 µM) under normoxia (20% O_2_) and hypoxia (0.1% O_2_). Activity levels were normalised to untreated controls and expressed as fold change. Data are shown as mean ± SD from three independent experiments (*** *p* < 0.001, **** *p* < 0.0001).

To investigate whether the cytotoxic effect of BU peptide was associated with apoptosis, caspase 3/7 activity was measured in COR-L24 and DMS79 cell lines under both normoxic and hypoxic conditions. Cells were treated with BU peptide, cisplatin, or etoposide at increasing concentrations for 24 h, and luminescence assays were performed to quantify caspase activation. As shown in Figure 7, BU peptide induced a significant, dose-dependent increase in caspase 3/7 activity in both cell lines, with a markedly greater response under hypoxia compared to normoxia. In contrast, treatment with cisplatin and etoposide resulted in only modest caspase activation, and their effects were comparable between normoxic and hypoxic conditions, showing no significant difference. The effect of BU peptide on caspase activation was notably stronger than that of cisplatin and etoposide, even at equivalent or higher concentrations. These findings suggest that BU peptide effectively induces apoptosis in SCLC cells and that this response is selectively enhanced under hypoxic conditions.

## 3. Discussion

SCLC is an aggressive neuroendocrine malignancy characterised by rapid progression, early metastasis, and high relapse rates following standard chemotherapy [1]. One of the major challenges in treating SCLC is the presence of hypoxic tumour microenvironments, which contribute significantly to therapy resistance and poor clinical outcomes [17]. Targeting hypoxia-adapted tumour cells, therefore, represents an important therapeutic strategy.

We previously synthesised the tryptophan analogue Boc-D-Trp(N-butyl)-OH and used it to prepare our pentapeptide via liquid-phase synthesis [11], though this approach was time-consuming. In this study, we developed solid-phase peptide synthesis (SPPS) using Boc chemistry, which offers faster assembly and better accessibility. In SPPS, temporary protecting groups are required to prevent alkylation of the indole ring during acidic Boc deprotection and cleavage conditions [18]. For the butyl-substituted tryptophan, we confirmed that the N^ind^-butyl modification remained stable throughout the synthesis and cleavage with hydrogen fluoride, as verified by the correct molecular mass of the final peptide. These findings indicate that the butyl group is chemically stable and compatible with Boc-SPPS, tolerating both 50% TFA and HF treatment. The successful removal of a formyl group with 20% piperidine in DMF, prior to cleavage from solid support further suggests that an Fmoc-D-Trp(N-butyl)-OH derivative could be synthesised for use in future SPPS applications.

GRPR is an attractive target for SCLC treatment due to its overexpression and implication of growth promoting effect, paralleled by a limited distribution in normal tissues [18,19]. While GRPR is known to be expressed under normoxic conditions [6], its regulation under hypoxia has not previously been characterised. In this study, we demonstrate for the first time that hypoxic exposure significantly upregulates GRPR protein expression in SCLC cell lines, DMS79 and COR-L24. This was further validated by increased binding of BBN-FITC, a well-established GRPR targeting ligand, under hypoxic conditions. Notably, DMS79 is a chemoresistant SCLC cell line, often used to model treatment-refractory disease for SCLC, making our findings particularly relevant to clinically resistant tumours where tumour hypoxia contributes to drug resistance. Our observations on hypoxia upregulated GRPR expression are consistent with previous studies showing that hypoxia can modulate G protein–coupled receptor (GPCR) expression in tumours. For instance, Park et al. demonstrated that hypoxia-inducible factor 1α (HIF-1α) mediates the transcriptional upregulation of the neuromedin B receptor (NMBR), a closely related bombesin receptor subtype, under hypoxic conditions in MDA-MB-231 breast cancer cells [10]. This parallel regulation underscores a broader role of hypoxia in modulating bombesin receptor family members, suggesting a potential role for GRPR in hypoxia-driven tumour cell survival and supporting its utility as a therapeutic target in anti-cancer strategies. While our findings confirm hypoxia-induced GRPR upregulation at the protein and functional levels, future studies will focus on evaluating HIF-1α–mediated transcriptional regulation of GRPR, which may further explain how hypoxia enhances GRPR expression and signalling in SCLC.

The cytotoxicity profile of BU peptide observed in this study highlights its potential as a promising therapeutic agent for SCLC, particularly in addressing hypoxia-induced treatment resistance. Conventional chemotherapeutic agents, such as cisplatin and etoposide, remain the standard of care for SCLC; however, our results indicate a differential sensitivity between cell lines. Specifically, DMS79 cells exhibited reduced sensitivity to cisplatin and etoposide compared to COR-L24 cells, suggesting an inherently more chemoresistant phenotype in DMS79. In contrast, BU peptide demonstrated superior cytotoxic potency at significantly lower concentrations, achieving greater reductions in cell viability than conventional agents. Importantly, BU peptide maintained, and even enhanced, its cytotoxic effect under hypoxic conditions in both COR-L24 and DMS79 cell lines. This observation suggests that BU peptide can potentially overcome hypoxia-induced resistance mechanisms, a major limitation associated with current chemotherapeutics. Notably, previous work from our group confirmed that related Bu-peptide analogues exhibited negligible cytotoxicity toward normal 3T3 fibroblast cells [12], supporting the tumour-selective activity of this peptide class. As BU peptide is derived from the SP-G sequence, its cytotoxic activity is closely linked to the expression of GRPR. Our findings are in good agreement with previous studies reporting that GRPR expression correlates with cellular sensitivity to SP-G inhibitors. For instance, HT29 colorectal adenocarcinoma cells, which express high levels of GRPR, exhibited increased sensitivity to SP-G inhibitors, whereas PANC-1 pancreatic carcinoma cells, characterised by low GRPR expression, demonstrated minimal responsiveness both in vitro and in vivo [16]. Additionally, GRPR expression has been implicated in modulating drug sensitivity profiles. In a study where CHO-K1 epithelial cells were transfected with the full-length human GRP receptor, GRPR-positive clones developed a transformed phenotype, displaying increased resistance to etoposide but enhanced sensitivity to substance P analogues, including SP-D and SP-G [20].

The ability of BU peptide to inhibit GRP-induced activation of downstream signalling pathways was demonstrated by its dose-dependent suppression of Akt and ERK1/2 phosphorylation in DMS79 cells. GRP stimulation markedly increased the phosphorylation of Akt at Ser473 and ERK1/2 at Thr202/Tyr204, consistent with the activation of the PI3K/Akt and MAPK/ERK pathways, which are known to promote tumour cell proliferation, survival, and resistance to apoptosis [20,21]. However, treatment with BU peptide, resulted in a significant reduction in phosphorylation levels of both Akt and ERK1/2, indicating effective blockade of GRP-GRPR-mediated signalling. This suggests that hypoxia-induced GRPR signalling may cooperate with HIF-1α–regulated survival pathways, further supporting GRPR as a critical mediator of tumour adaptation under low-oxygen conditions.

The competitive binding study further validated the ability of the BU peptide to directly interact with GRPR. This observation aligns with findings by Orosz et al. (1995) [22], where short-chain substance P antagonist analogues demonstrated specific GRPR binding. In our study, BU peptide induced a reduction in BBN-FITC fluorescence intensity, indicative of receptor competition. This effect was more pronounced under hypoxic conditions, consistent with elevated GRPR expression levels. It is important to note that BU peptide is structurally derived from the SP-G analogue, originally designed as a substance P antagonist. Given that BU peptide shares almost no sequence homology with bombesin, similar to SP-G, its binding affinity to GRPR relative to bombesin may be comparatively low. Consequently, FITC-bombesin may displace GRPR bound BU peptide, potentially accounting for the residual BBN-FITC signal observed. As a derivative of SP-G, BU peptide likely functions as a broad-spectrum neuropeptide antagonist, with potential activity across multiple GPCRs, including vasopressin (V1A), bradykinin (BK2), and GRPR [16]. To confirm receptor selectivity and binding parameters, more comprehensive mechanistic studies targeting individual receptors are warranted.

We also evaluated the mode of cell death by investigating the ability of the BU peptide to induce caspase 3/7 activation. Caspase-3 and 7 are known to play a central role in the execution of apoptosis [23]. Caspase-3 is known for its role in cleaving a wide variety of cellular substrates and promotes DNA fragmentation, both of which lead to cell death [23]. Caspase-3 can be activated through caspase-8 and caspase-9 by extrinsic or intrinsic signalling, respectively [24]. In our data, BU peptide treatment led to a significant, dose-dependent increase in caspase-3/7 activity in both COR-L24 and DMS79 cells, with a notably stronger apoptotic response under hypoxic conditions. This selective enhancement of apoptosis in hypoxia suggests that BU peptide can effectively target hypoxia-adapted tumour cells, which are typically resistant to conventional chemotherapeutic agents. In contrast, cisplatin and etoposide induced only modest caspase activation, with no significant difference between normoxic and hypoxic conditions, consistent with their primary mechanisms of action through DNA damage rather than direct apoptotic pathway activation [25,26]. Furthermore, the limited caspase response to these standard agents in DMS79 cells aligns with their known chemoresistant phenotype, underscoring the need for alternative therapeutic strategies capable of inducing apoptosis in resistant tumour populations. However, the exact apoptotic mechanism by which BU peptide works is not known. BU peptide is derived from SP-G, which is a novel class of anti-cancer agent that inhibits SCLC cell growth in vitro and in vivo [27,28]. Previous reports have found that SP-G induces cell death and apoptosis by stimulating c-jun-N-terminal kinase (JNK) activity [29].

Together, our study provides compelling evidence that BU peptide antagonises GRPR by competitively blocking GRP-induced receptor activation and downstream PI3K/Akt and MAPK/ERK signalling, thereby inducing apoptosis and suppressing tumour cell viability. These findings demonstrate that BU peptide exploits a hypoxia-induced vulnerability in SCLC, establishing GRPR as a hypoxia-inducible therapeutic target. The results also position BU peptide as a promising candidate for further translational development, particularly for overcoming hypoxia-driven resistance associated with conventional chemotherapy. Peptide-based therapeutics, such as the BU peptide, are generally well tolerated in vivo and offer promising potential for systemic targeted delivery. BU peptide, like its precursor SP-G, has demonstrated good plasma stability and has been shown to induce tumour regression in xenograft models [12,30,31], supporting its therapeutic relevance. For clinical translation, the short and amphipathic nature of BU peptide’s amino acid sequence makes it particularly amenable to advanced drug delivery strategies, such as liposomal encapsulation. This approach not only enhances peptide stability and bioavailability but also mitigates potential off-target toxicity. Moreover, liposomal formulations can improve tumour-specific accumulation via enhanced permeability and retention (EPR) effects, a key advantage in solid tumour targeting. Given its broad-spectrum activity and favourable pharmacokinetic profile, BU peptide represents a strong candidate for further development as a multi-targeted therapeutic agent.

Overall, this study supports the advancement of GRPR-targeted peptide therapeutics as a novel and versatile strategy for treating aggressive cancers such as SCLC.

## 4. Materials and Methods

### 4.1. Cell Lines and Culture Conditions

Small cell lung cancer cell lines COR-L24 and DMS79 were obtained from Professor Anne White (Faculty of Life Sciences, The University of Manchester). Cells were cultured in RPMI-1640 medium (Gibco, Thermo Fisher Scientific, Waltham, MA, USA) supplemented with 10% foetal bovine serum (FBS; Sigma-Aldrich, St. Louis, MO, USA). Both lines were routinely tested for mycoplasma contamination and authenticated in-house using short tandem repeat (STR) profiling at the University of Manchester Core Facility. Cells were maintained at 37 °C in a humidified incubator with 5% CO_2_. Normoxic culture conditions were defined as 20% O_2_, while hypoxia was achieved using a hypoxia chamber (Don Whitley Scientific, Bingley, UK) set to 0.1% O_2_, 5% CO_2_, and balanced N_2_. Cells were pre-conditioned under normoxia or hypoxia for 24 h before drug treatment or sample collection. DMS79 cells were originally established from the pleural fluid of a patient with small-cell lung carcinoma treated with cytoxan, vincristine, methotrexate, and radiotherapy [32]. COR-L24 cells were established from a lymph node biopsy of an untreated 71-year-old male diagnosed with SCLC [33].

### 4.2. Peptide Synthesis and Characterisation

The BU peptide (DMePhe-DTrp-Phe-DTrp(N-butyl)-Leu-NH_2_) was synthesised manually by solid-phase t-Boc chemistry using 0.5 mmol p-methylbenzhydrylamine (MBHA) resin. The analogue Boc-D-Trp(N-butyl)-OH was prepared as described previously [11]. Amino acid coupling was performed with 3 molar equivalents of Boc-protected amino acid and 3 molar equivalents of the coupling reagent N,N,N′,N′-tetramethyl-O-(1H-benzotriazol-1-yl)uronium hexafluorophosphate (HBTU; Iris Biotech, Marktredwitz, Germany) dissolved in DMF. To this, 9 molar equivalents of N,N-diisopropylethylamine (DIPEA; Sigma-Aldrich, St. Louis, MO, USA) were added, and reactions were allowed to proceed for 40 min. Successful coupling was confirmed by the Kaiser test [34]. Peptide cleavage from the resin was performed with hydrogen fluoride (HF) at 4 °C in the presence of p-cresol and thiocresol (Sigma-Aldrich) as scavengers. The crude peptide was precipitated and washed with cold diethyl ether, solubilised in 20% *v/v* acetic acid in water, and lyophilised. Purification was achieved by reverse-phase HPLC on a C4 column (ACE 10C4, 250 × 21.2 mm i.d.; Advanced Chromatography Technologies, Aberdeen, UK). Mass spectrometry confirmed the expected molecular ion.

### 4.3. Western Blot Analysis

GRPR protein expression was assessed by Western blot under normoxic and hypoxic conditions. Cells were lysed in RIPA buffer (Thermo Fisher Scientific) containing protease and phosphatase inhibitors. Protein concentrations were quantified using a BCA assay (Thermo Fisher Scientific). Equal protein amounts (20 µg) were separated by SDS-PAGE and transferred onto PVDF membranes (Millipore, Burlington, MA, USA). After blocking in 5% milk in TBST, membranes were incubated overnight at 4 °C with primary antibody against GRPR (1:1000; Abcam, Cambridge, UK). β-Actin (1:5000; Santa Cruz Biotechnology, Dallas, TX, USA) was used as a loading control. HRP-conjugated secondary antibodies (Abcam) were applied, and bands were visualised using ECL substrate (Bio-Rad, Hercules, CA, USA). Band intensities were quantified using ImageJ software,version 1.53a (National Institutes of Health, Bethesda, MD, USA) and normalised to β-actin as the internal loading control and normalised to β-actin as the internal loading control.

### 4.4. Immunofluorescence Staining

COR-L24 and DMS79 cells were seeded on Ibidi µ-Slides (Ibidi, Martinsried, Germany) and incubated under normoxia (20% O_2_) or hypoxia (0.1% O_2_) for 24 h. Cells were fixed with 3.5% formalin, blocked with 1% BSA, and incubated overnight at 4 °C with anti-GRPR antibody (1:200; Antibodies.com, Cambridge, UK). Alexa Fluor 488-conjugated secondary antibody (Thermo Fisher Scientific) was used for detection, and nuclei were counterstained with DAPI (1 µg/mL). Images were acquired using a 40×/0.80 Plan Apo objective on a 3DHistech Panoramic 250 Flash II slide scanner. ImageJ software (NIH, Bethesda, MD, USA) was used for analysis.

### 4.5. Flow Cytometry for GRPR Binding

DMS79 cells were cultured under normoxia or hypoxia for 24 h, then incubated with bombesin-FITC (BBN-FITC; Peptide Protein Research Ltd., Fareham, UK) at 3, 5, or 10 µM for 30 min at room temperature. Cells were washed with PBS and analysed on a BD LSRFortessa X20 flow cytometer (BD Biosciences, San Jose, CA, USA). Data were gated to exclude debris and doublets, and only viable single cells were included in the analysis. Mean fluorescence intensity (MFI) values were calculated using FlowJo software, version 10.10 (BD Biosciences, San Jose, CA, USA).

### 4.6. Capillary-Based Immunoassay (Abby System)

DMS79 cells were seeded in 6-well plates and incubated under hypoxic conditions for 24 h to induce GRPR expression. Cells were then serum-starved for 4 h, pre-treated with BU peptide (0–50 µM) for 30 min, and stimulated with GRP (100 nM; Sigma-Aldrich) for 15 min. After treatment, cells were washed with ice-cold PBS and lysed in RIPA buffer containing protease and phosphatase inhibitors. Protein concentrations were determined by BCA assay. Samples were analysed using the Abby automated capillary Western bot system (ProteinSimple, Bio-Techne, Minneapolis, MN, USA) according to the manufacturer’s instructions. Primary antibodies used included GRPR (Abcam), phospho-Akt (Ser473; CST #9271, 1:50), total Akt (CST #9272, 1:50), phospho-ERK1/2 (Thr202/Tyr204; CST #9101, 1:50), total ERK1/2 (CST #9102, 1:50), and β-actin (CST #4967, 1:100). HRP-conjugated secondary antibodies and chemiluminescent reagents were supplied in the Detection Module. Data were quantified using Compass software, version 6.1.0 (ProteinSimple, San Jose, CA, USA). Phosphorylated Akt and ERK1/2 signals were quantified using Compass software (ProteinSimple) and normalised to their corresponding total Akt and ERK1/2 levels.

### 4.7. Competitive Binding

The competitive binding study of BU peptide to GRPR with BBN-FITC was performed using flow cytometry. DMS79 cells were pre-incubated under normoxic and hypoxic conditions for 24 h to induce differential GRPR expression. Cells were then treated with BU peptide (5–10 µM) for 1 h, followed by incubation with BBN-FITC (10 µM), a fluorescent GRPR ligand, for 30 min at 37 °C in the dark. After washing twice with cold PBS, fluorescence intensity was analysed using a BD LSRFortessa flow cytometer (BD Biosciences) and quantified using FlowJo software (v10). Mean fluorescence intensity (MFI) values were normalised to the untreated control.

### 4.8. Cell Viability Assay

Cell viability was measured using the CellTiter-Glo assay (Promega, Madison, WI, USA). COR-L24 and DMS79 cells were seeded into 96-well plates. Cells were then pre-conditioned either under normoxia (20% O_2_) or hypoxia (0.1% O_2_) for 24 h, then returned to normoxia for treatment. Cells were exposed to BU peptide (0–20 µM), cisplatin (0–150 µM; Sigma-Aldrich), or etoposide (0–150 µM; Sigma-Aldrich) for 24 h. CellTiter-Glo reagent was added, incubated for 10 min, and luminescence was recorded on a Synergy H1 microplate reader (BioTek Instruments, Winooski, VT, USA).

### 4.9. Caspase 3/7 Activity Assay

Apoptosis was assessed using the Caspase-Glo^®^ 3/7 assay (Promega, Madison, WI, USA) according to the manufacturer’s instructions. Cells were seeded in 96-well plates and treated with BU peptide, cisplatin, or etoposide under normoxic and hypoxic conditions. After 24 h, Caspase-Glo reagent was added, and luminescence was measured 1 h later using a Synergy H1 reader (BioTek Instruments, Winooski, VT, USA).

### 4.10. Statistical Analysis

All experiments were performed in three independent biological experiments. Data are presented as mean ± standard deviation (SD). Statistical analyses were performed in GraphPad Prism v10 (GraphPad Software, San Diego, CA, USA). For multiple comparisons, one-way ANOVA followed by Tukey’s post hoc test was used. For two-group comparisons, unpaired two-tailed Student’s *t*-tests were applied. Statistical significance was defined as follows: *p* < 0.05 (*), < 0.01 (**), < 0.001 (***), < 0.0001 (****).

## 5. Conclusions

This study highlights the potential of a novel butylated neuropeptide antagonist, BU peptide, as a targeted therapeutic agent for SCLC, particularly in the context of hypoxia-induced treatment resistance. We provide the first direct evidence that GRPR is upregulated under hypoxic conditions in SCLC cells and demonstrate that BU peptide effectively targets this hypoxia-associated vulnerability. BU peptide not only inhibits GRPR-mediated activation of pro-survival signalling pathways, PI3K/AKT and MAPK/ERK, but also induces potent, apoptosis-driven cytotoxicity effects that are significantly enhanced in hypoxic environments. Compared to conventional chemotherapies such as cisplatin and etoposide, BU peptide exhibited superior potency at lower concentrations and greater efficacy in hypoxia-adapted cells. These findings support the continued development of GRPR-targeted peptide therapeutics and provide a strong rationale for advancing BU peptide toward in vivo evaluation and clinical translation for the treatment of refractory and hypoxic SCLC.

## Data Availability

The data generated during this study are available by request to the corresponding author.
